# Complex patch geometry promotes species coexistence through a reverse competition–colonization trade-off

**DOI:** 10.1098/rspb.2023.1554

**Published:** 2023-11-01

**Authors:** Nina Rynne, Geneva Birtles, Jamie Bell, Mung Suan Pau Duhlian, Samuel McNeil, Adel Mehrpooya, Blake Noske, Yadursha Vakeesan, Michael Bode

**Affiliations:** ^1^ School of Mathematical Sciences, Queensland University of Technology, 4 George Street, Brisbane, Queensland 4000, Australia; ^2^ School of Mathematics and Applied Statistics, University of Wollongong, Wollongong, New South Wales 2522, Australia; ^3^ School of Science, Royal Melbourne Institute of Technology, Melbourne, Victoria 3001, Australia; ^4^ College of Engineering, Science and Environment, The University of Newcastle, Newcastle, New South Wales 2300, Australia

**Keywords:** coexistence theory, species competition, dispersal, landscape, niche, mechanism

## Abstract

Explaining the maintenance of diverse species assemblages is a central goal of ecology and conservation. Recent coexistence mechanisms highlight the role of dispersal as a source of the differences that allow similar species to coexist. Here, we propose a new mechanism for species coexistence that is based on dispersal differences, and on the geometry of the habitat patch. In a finite habitat patch with complex boundaries, species with different dispersal abilities will arrange themselves in stable, concentric patterns of dominance. Species with superior competitive and dispersal abilities will dominate the interior of the patch, with inferior species at the periphery. We demonstrate and explain the mechanism on a simple one-dimensional domain, and then on two-dimensional habitat patches with realistic geometries. Finally, we use metrics from landscape ecology to demonstrate that habitat patches with more complex geometries can more easily support coexistence. The factors that underpin this new coexistence mechanism—different dispersal abilities and habitat patches with complex geometries—are common to many marine and terrestrial ecosystems, and it is therefore possible that the mechanism is a common factor supporting diverse species assemblages.

## 1. Introduction

Understanding the mechanisms that maintain species coexistence is a central question in ecology: critical for understanding biodiversity, and also for helping to conserve it [1–3]. Traditional mechanisms for coexistence are based on differences between the coexisting species. These include their partitioning of local resources [4], differential exposure to and recovery from disturbance [5,6] and different responses to varying environmental conditions [3].

Spatial heterogeneity in the environment can also support coexistence [7,8]. When resources or conditions vary in space, different species can achieve local dominance in different subregions (through environmental filtering), and source–sink dynamics can allow them to co-occur locally [9]. Spatial storage effects [2] and competition–colonization trade-offs [10] allow species to increase from low abundance by exploiting transient temporal or spatial variation. Even in the absence of variation—that is, even when the environment is spatially and/or temporally homogeneous—species can coexist when their dispersal abilities differ in particular ways, or where different species disperse through space upon different networks [11–13].

Stable patterns of coexistence can also be created by an interaction between the geometry of spatial resources (i.e. the location of habitat patches), and species' ability to move between those resources [14]. Different species can establish dominance in regions with particular geometric properties—high densities of habitat patches, for example [15,16]—because their dispersal abilities are well suited to those properties. Previously identified geometric mechanisms of species coexistence emerged from multispecies metapopulation theory, particularly in patchy marine environments. As a natural consequence, they all considered geometric variation in the distribution of discrete habitat patches, both theoretically and empirically [14,15,17–19]—that is, the geometry of inter-patch distances. Coexistence resulted from the different ways that species moved between patches.

By contrast, in this paper we propose a geometric mechanism for species coexistence that is based on the way different species move within a single habitat patch—that is, the geometry of intra-patch distances. The geometry of ecological landscapes is complex and highly diverse [20,21]. Habitat loss and fragmentation has led to an interest in understanding how geometric coexistence mechanisms exploit variation in the size and spacing of habitat patches at a regional scale to promote coexistence. If ecological processes can transform geometric diversity into species diversity, this would represent a plausible, common and intuitive explanation for high levels of species coexistence. In this mechanism, coexistence emerges from an interaction between species' different competitive and dispersal abilities, and variation in the geometry of the patch boundary—that is, variation in the shape of the habitat patch itself.

Specifically, we propose that species with inferior competitive abilities—that is, species that would normally be excluded from a community—can persist at the boundaries of finite habitat patches. Their lower dispersal abilities would normally be a limitation in a classic competition–colonization trade-off because higher dispersal abilities allow species to find and occupy temporarily vacant sites. However, when habitat patches are finite, a higher dispersal ability carries a proportion of the offspring into the surrounding unsuitable habitat, and is therefore a source of mortality (or emigration).

We begin by demonstrating how our mechanism allows two species to coexist in a single, bounded, one-dimensional habitat patch. We use this simple example to elucidate precisely how the shape and location of habitat boundaries (i.e. the geometry of the patch) allow species that would normally exclude each other to coexist. Then, we use a set of real-world geometries—specifically, the shapes of Western Australian islands—to demonstrate that multiple species can coexist in complex, bounded landscapes. Finally, we use metrics from landscape ecology to investigate what types of geometric complexity are responsible for supporting coexistence.

## 2. Coexistence in a one-dimensional landscape

We start by considering the changing abundance of two competing populations, *u*_1_ and *u*_2_, through time *t*, that disperse randomly along the *x*-dimension—a homogeneous and bounded linear habitat patch. Following Gopalsamy [19], we describe this situation with two coupled reaction–diffusion partial differential equations (PDEs):

2.1 $$\begin{array}{rl} \frac{\partial u_{1}}{\partial t} & =D_{1}\frac{\partial^{2}u_{1}}{\partial x^{2}}+u_{1}(r_{1}-a_{11}u_{1}-a_{12}u_{2})\\ \mathrm{and}\;\frac{\partial u_{2}}{\partial t} & =D_{2}\frac{\partial^{2}u_{2}}{\partial x^{2}}+u_{2}(r_{2}-a_{22}u_{2}-a_{21}u_{1}). \end{array}\}$$

Reaction–diffusion PDE models have been used to describe organisms that move throughout their lifetimes [22], and also organisms that disperse once (e.g. marine organisms with sedentary adults and natal dispersal [23]). When formulated as a one-dimensional PDE, we effectively assume that some proportion of organisms stay in their natal location, and the remaining proportion move either to the immediate left or to the immediate right. The diffusion coefficients *D_i_* reflect each species' tendency to move rather than stay: higher values of *D_i_* indicate that a larger proportion move. The parameters *r_i_* denote each population's intrinsic growth rate, and *a_ij_* indicate the *per capita* effect of species *j* on individuals of species *i*, with species interactions only occurring locally (i.e. with those individuals who co-occur at location *x*).

We initialize the system such that the two species have equal, uniform and low abundance: *u*_1_(*x*, 0) = *u*_2_(*x*, 0) = *u*_0_. We assert Dirichlet boundary conditions: *u_i_*(0, *t*) = *u_i_*(1, *t*) = 0, indicating that the patch is surrounded by habitat that is unsuitable for the species, such as the coastline of an island for terrestrial species. These boundary conditions suggest that the organisms will move across the boundary if they encounter it, thereby exiting the habitat (and the population). This makes our model applicable to species with dispersal that is either initially passive (e.g. marine organisms with a passive precompetent dispersal phase [24]) or completely passive (e.g. seeds or spores [25]).

**Figure 1.**
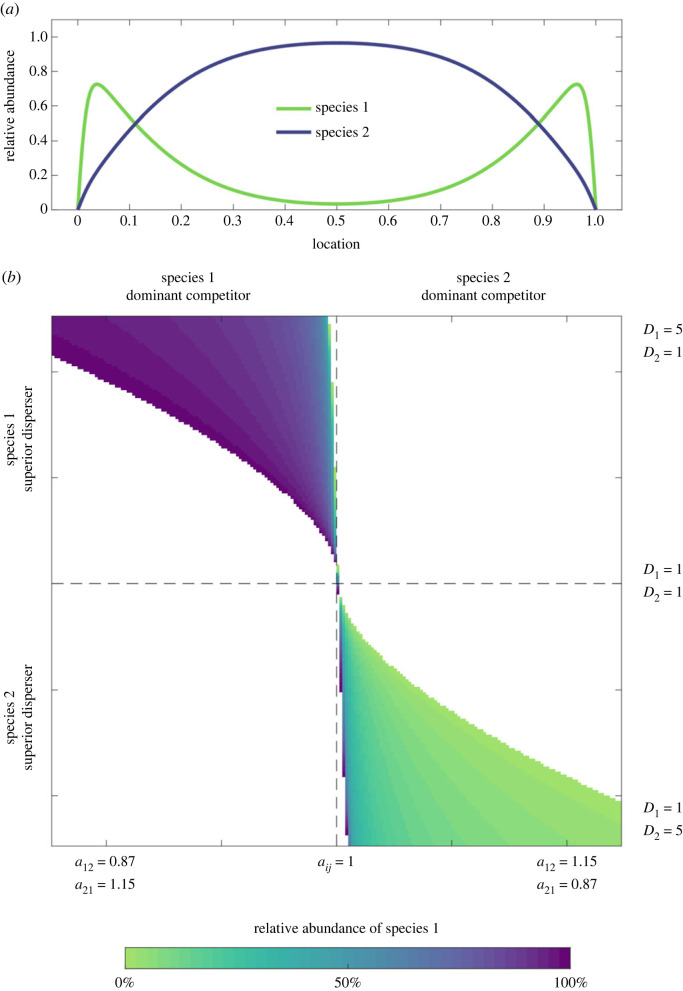
Spatial coexistence of two species on a linear bounded habitat patch. (*a*) The abundance of the two species, simulated to equilibrium. Species 1 is both a superior competitor and a superior disperser and dominates the interior of the patch. Species 2 is an inferior competitor and disperser, and yet is able to dominate at the boundaries of the patch. (*b*) Range of competitive and dispersal abilities that allow the two species to stably coexist. Note that the values of *D* are scaled by 10^−4^. See electronic supplementary material, tables S1 and S2 for the parameter values.

To demonstrate that our mechanism can generate stable coexistence, we will simplify the model by assuming that the intrinsic growth rates of the species are equal (*r_i_* = *r*), that density-dependent terms are unity (*a_ii_* = 1), that species interactions are inversely symmetric (i.e. *a_ij_* = (*a_ji_*)^−1^), and that species 1 is the dominant species (*a*_21_ > 1). Gause's competitive exclusion principle [26] would thus expect species 1 to exclude species 2. In the non-spatial version of this model, this is in fact what occurs. However, we also assume that the competitively dominant species is also a better disperser (i.e. *D*_1_ > *D*_2_). When we simulate this system of PDEs to equilibrium (defined by total absolute changes of less than 10^−6^, over one time step), we find that this combination of parameters allows coexistence to occur (figure 1*a*; electronic supplementary material, figure S1). Figure 1*b* shows a range of parameters across which coexistence is possible. See Data accessibility for a link to the code, and the electronic supplementary material for the simulated dynamics reaching equilibrium.

As shown in figure 1*a*, this coexistence mechanism generates stable coexistence with varying spatial dominance, symmetric around the midpoint of the linear habitat patch. Far from the boundaries, the competitive dominant (species 1) has the greater abundance, and is often able to exclude the inferior species (as in figure 1*a*). At the patch boundaries, however, the competitive inferior species becomes the more abundant, although it is never able to exclude the competitive dominant from any location. Equation (2.1) therefore allows stable coexistence, via a modified competition–colonization trade-off. Unlike the classic formulation of competition–colonization coexistence [10], in our mechanism a lower dispersal ability confers a competitive advantage near the boundary. Because organisms that cross the boundaries are lost to their populations, higher dispersal results in higher mortality. It becomes locally advantageous to have lower dispersal abilities near the boundary of a habitat patch.

Two other conclusions can be drawn from figure 1*b*. First, coexistence is not possible along the vertical line when *α_ij_* = *α_ji_*. Under these competitively neutral conditions, the inferior disperser excludes the superior—that is, dispersal differences alone are not enough to deliver coexistence in our model. Second, our mechanism cannot generate the type of coexistence seen in the traditional competition–colonization trade-off. This trade-off occurs in the upper-right and lower-left quadrants of figure 1*b*, where coexistence is not observed.

## 3. Coexistence in two-dimensional geometries

The process that generates coexistence in our one-dimensional model is relatively intuitive, and produces comprehensible patterns of species abundance. Coexistence is based on stable geographical patterns of replacement, with inferior competitors able to persist at the periphery of the habitat patch, where their competitive inferiority is offset by lower dispersal-driven mortality rates. However, in two-dimensional landscapes with realistic geometries, ‘proximity' to boundaries becomes more complicated.

We begin our two-dimensional analyses by extending our model to *S* species, competing within the bounds of an irregular, two-dimensional habitat patch *Ω*:

3.1 $$\frac{\partial u_{i}}{\partial t}=D_{i}\Delta u_{i}+u_{i}\left(r_{i}-\overset{S}{\underset{\;j=1}{\sum }}a_{ij}u_{j}\right),\;\mathrm{for}\;i=1,\;\ldots ,\;S.$$

The boundary conditions are again Dirichlet: *u_i_*|_∂*Ω*_ for all *i* = 1, … , *S*; the initial conditions are again that all species are present at low abundance: *u_i_*(*x*, 0) = *u*_0_ for all *x* ∈ *Ω*. We again use a numerical solver to simulate the dynamics of each population, using forward-time-centred-space methods applied at a fine (but computationally affordable) spatial resolution. See 'Data accessibility' for a link to the code.

Based on our one-dimensional results, we expect coexistence to occur when a competitive hierarchy is matched by a dispersal hierarchy. To test whether this result would be repeated in two dimensions, we simulate the dynamics of randomly generated sets of species, on patches of habitat with complex geometries. To give our patches realistic shapes, we digitize satellite images of coastal islands from the Kimberley archipelago in Western Australia. Selecting three islands from the archipelago, we simulate competition among four species as described in equation (3.1). The parameters of this competitive system—the off-diagonal elements of the interaction matrix **A**, the shared intrinsic growth rate *r*, and the vector of dispersal abilities $D=\{D_{1},\;\ldots ,D_{4}\}$—are lognormally distributed random variables. For each island, we generate random, four-species competitive ecosystems, simulate their dynamics to equilibrium (defined by total absolute changes of less than 10^−6^, over one time step), and repeat this procedure until we find a combination of random species where all four can coexist.

**Figure 2.**
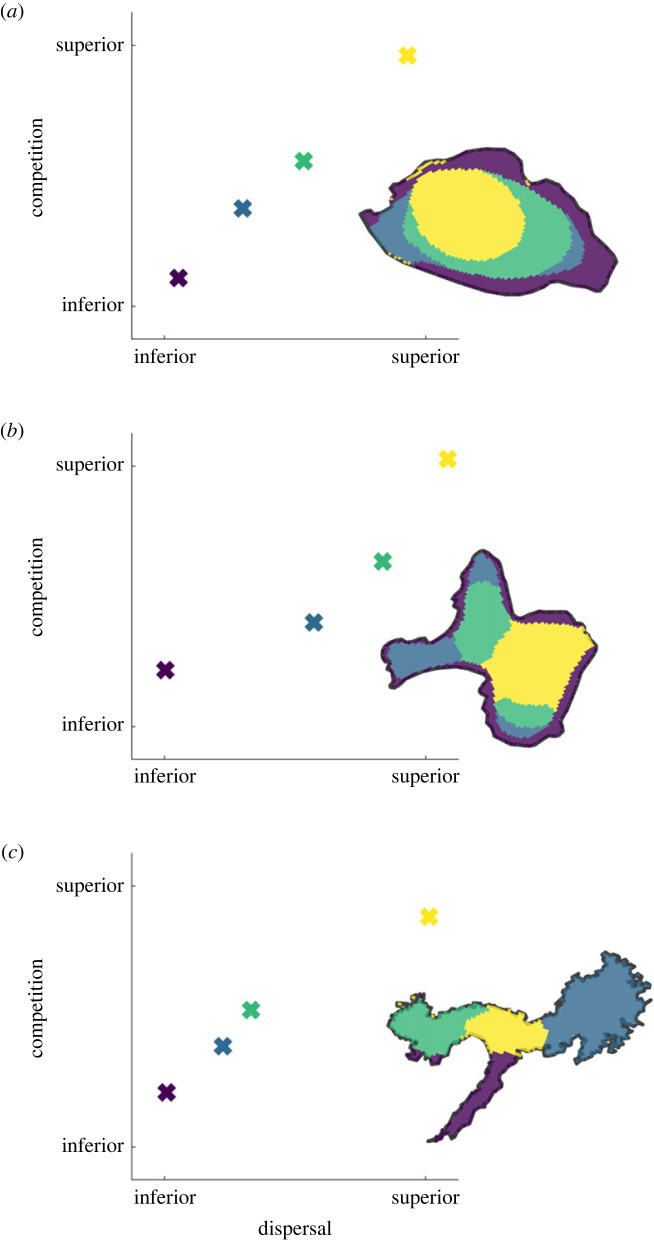
Spatial coexistence of four species on islands with different geometries. In each panel, we show a bivariate plot of each species' competitive ability against its dispersal ability. We also produce a map of each island, with the dominant species at each point in space denoted by its corresponding colour. All values are shown at equilibrium, calculated by simulation. See electronic supplementary material, tables S3–S5 for the parameter values for each panel.

Figure 2 shows the successful results of this random search, for each of the three islands. See 'Data accessibility' for a link to the code, including the parameter sets used for each island. Each panel shows an image of an island, with colours (yellow, green, light blue, dark blue) indicating which species is most abundant at each point in space. In these results, we see echoes of the spatial patterns of dominance observed in one-dimension, with particular species dominating close to the boundaries, and different species dominating the centre of the islands. This is particularly true for figure 2*a*, which has the simplest geometry. However, in figure 2*b,c*, multiple species dominate boundary locations, and more than one species dominates a location that could be reasonably described as ‘interior'.

When we produce bivariate plots of the species' competitive and dispersal abilities, it becomes apparent that we are again observing the same competition–colonization trade-off seen in the one-dimensional model (figure 2). In each panel, we plot the dispersal ability of each species *D_i_* on the *x*-axis, and its competitive ability on the *y*-axis. Specifically, we plot the total impact of species *i* on all other species: $y_{i}=\sum_{j}a_{\;ji}$, such that large values correspond to superior aggregate competitors. In each case, we see that coexistence occurs when superior competitors are also superior dispersers.

## 4. Geometric complexity and biodiversity

For each of the islands in figure 2, a different combination of four species are able to coexist. Finding a set of species for each island requires repeated searches of parameter space (specifically, repeated random choices of dispersal abilities *D_i_* and growth rates *r_i_*). In the process of this search, we observed a counterintuitive result: the island with simplest geometry (figure 2*a*) requires more searches than the island whose geometry is the most complex (figure 2*c*). On the basis of this observation, we hypothesize that coexistence is easier to achieve on habitat patches with more complex geometries. To investigate whether this is true, we first need to define what we mean by the ‘difficulty' of achieving coexistence, and by the ‘complexity' of a patch's geometry.

### (a) Ease of coexistence

Inspired by the random approach we use to identify the sets of coexisting species in figure 2, we define coexistence as ‘easy' if randomly generated species have a high probability of stable coexistence. By contrast, we define coexistence as ‘difficult' if randomly generated species have a low probability of stable coexistence. For a given habitat patch, we therefore generate *S* = 4 randomly parameterized species, and introduce them all simultaneously to a vacant habitat patch, at low abundance. We simulate the ecosystem dynamics until the species reach numerical equilibrium. We then record the number of coexisting species (defined as *n_i_* > 0.05 of maximum abundance) remaining. We repeat this procedure for 850 random ecosystems, and calculate the average number of species that coexist on the habitat patch. High average numbers of persisting species (e.g. *S* ≈ 4) indicate that coexistence is easy, while low numbers (e.g. *S* ≈ 1) indicate that coexistence is difficult.

### (b) Geometric complexity

There are many ways to define the complexity of a planar polygon. Contributing factors include the number of vertices, the concavity and its fractal geometry. Of all the metrics available [27], we chose two metrics that measure very different elements of geometric complexity. The first metric *m*_1_ measures the complexity of the overall habitat patch, by calculating the difference between the perimeter of the patch and the perimeter of a hypothetical circular patch with equal area. Generally speaking, patches with high values of *m*_1_ contain macro-scale structures such as peninsulas and inlets, and alternating concave and convex regions. The second metric *m*_2_ measures the small-scale complexity of the habitat patch, using Kolmogorov complexity. This metric measures the frequency and magnitude of angular changes, as the perimeter of the shape is traversed [28]. It takes high values on patches with jagged perimeters, even if the overall shape of the patch remains relatively regular. See 'Data accessibility' for a link to code that calculates these two metrics for a given habitat patch outline.

**Figure 3.**
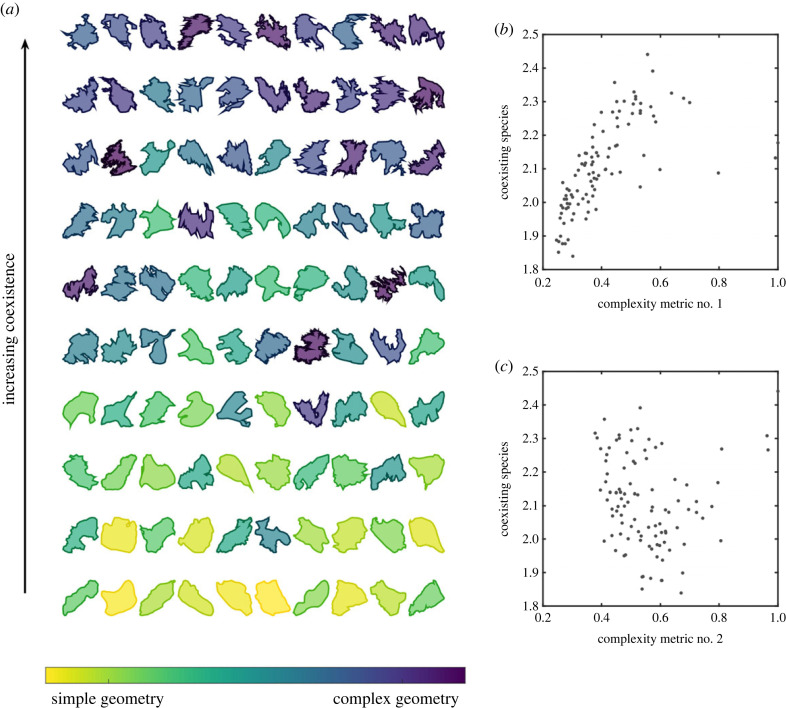
Relationship between an island's geometric complexity, and the number of randomly generated species that can coexist on those islands. (*a*) The outline of each island, ordered by the average number of species that can coexist at equilibrium (calculated by simulation). Islands are coloured by their geometric complexity, as measured by *m*_1_. (*b*,*c*) Scatterplots of the average number of coexisting species (*y*-axis), against our two different metrics of geometric complexity. Metric no. 1 measures macro-scale complexity, and is positively correlated (*r* = 0.76) with the average number of coexisting species. Metric no. 2 measures fine-scale complexity, and is negatively correlated (*r* = −0.27) with the average number of coexisting species. See 'Data accessibility' for all parameter values.

To test the relationship between our metrics of geometric complexity and coexistence, we calculate the values of both metrics for the 100 largest islands of the Kimberley archipelago. Figure 3 shows the outlines of these islands, and compares their geometric complexity with the ease of coexistence. See 'Data accessibility' for a link to the code that creates this figure. The results show that only one of our complexity metrics is positively correlated with coexistence—the island-wide metric of complexity *m*_1_ (*r* = 0.76). There is a fairly strong association between islands with more complex macro-scale structures, and islands that can easily support coexistence. By contrast, our metric of small-scale complexity, *m*_2_, has a low negative correlation (*r* = −0.27) with coexistence. The fine-scale shape of the boundary does not appear to predict whether species can coexist in a habitat patch.

## 5. Discussion

In an inversion of the classic competition–colonization coexistence mechanism, we show that species that are inferior in both their competitive and dispersal abilities can readily coexist alongside superior species, in a finite patch with a complex boundary. The processes that generate the proposed mechanism are found across most terrestrial and marine ecosystems: a habitat patch with irregular geometry surrounded by unsuitable habitat, and species with differing dispersal abilities. It is possible that the coexistence mechanism we identify is also common.

Our coexistence mechanism offers an interesting new perspective on coexistence. Gause's Law of competitive exclusion, in its most general form, holds that species must differ in some important way if they are to coexist. Our analyses show that the naturally complex geometry of habitat patches could be a source of those differences—essentially, species can persist and locally dominate if they are able to find a suitable geometric niche. A diversity of species could therefore be partly the result of a diversity of complex landscape geometries, interacting with species' different dispersal abilities.

Most competition–colonization trade-offs produce ephemeral patterns of species dominance; by contrast, our mechanism produces predictable spatial patterns of species dominance. At its most straightforward, species dominate in concentric circles, with superior competitors most abundant in the interior of habitat patches, while inferior competitors dominate the boundaries, where dispersal becomes a liability. Irregular geometry, such as the shapes seen in natural habitat patches, can complicate this simple dominance pattern. In the concave island outlines we analysed in figure 2*c*, for example, both competitively dominant and subordinate species can be found at the habitat boundary. Species dominance is often associated with large-scale geometric features—peninsulas for example, or concave pockets of unsuitable habitat. More complicated habitat geometries could offer more opportunities for species to carve out unique geometric niches. Indeed, we found that more complex habitat geometries could more readily support coexistence.

Our mechanism has potential consequences for spatial conservation planning. Landscape ecology has long held that edge effects are detrimental to species persistence [29], and conservation planning therefore seeks to create large, compact protected areas. For example, spatial prioritization tools use boundary length modifiers to automatically create regular, convex geometries [30]. Human land-use patterns also produce rectilinear habitat patches that are more regular than natural patches [27], following roads, latitudes or cadastral boundaries, and therefore less geometrically complex. Our mechanism adopts this standard view of edge effects—as sources of mortality—but we find that the implications can be positive for particular species, and therefore conducive to coexistence. Edges have a negative demographic impact on populations, but if this negative impact differs between species, then edges can help support species diversity. There may therefore be benefits to creating or maintaining concave, irregular habitat boundaries, particularly in areas where fragmentation occurs naturally.

While complicated, our multispecies models (equations (2.1) and (3.1)) are simplifications of real ecological communities. PDE dynamics are deterministic and continuous, and do not account for the stochastic and spatio-temporally discrete nature of real ecosystems. The absence of stochasticity is particularly important to acknowledge, given the important role it can play in coexistence [3,31], particularly where dispersal is common [11,16] The functional form of the PDEs—their reaction, diffusion and interaction components—are assumptions; different choices would result in different outcomes, and might affect our coexistence results. For example, our models assume a diffusive form of dispersal, but many ecological models use dispersal kernels [32,33]. However, while kernel dispersal has some impact on the equilibrium abundance of the species, the same basic pattern of spatial dominance still results (electronic supplementary material, figure S2). Finally, our analyses do not consider whether or how coexisting species could have evolved the interaction dynamics required to allow coexistence—that is, the evolutionary stability of the mechanism—a common and important limitation of many coexistence theories [34]. Each of these assumptions is standard, however, and a simple, standard model allows us to deliver on our primary goal—to illustrate a new coexistence mechanism, and to show that it could lead to stable coexistence.

Illustrating that a mechanism can theoretically lead to coexistence does not imply that it necessarily plays a role in maintaining species diversity in real communities. While we do not provide empirical evidence to support our mechanism, we note that previous empirical research into competition–colonization trade-offs has revealed ambiguous relationships between dispersal and competitive abilities [35–37], and sometimes weakly positive relationships (as required by our mechanism; [38]). Rather than supporting either type of competition–colonization trade-off, these results emphasize the challenges in measuring and comparing multiple traits in real ecological settings, and thus in providing empirical support for any individual low-dimensional coexistence mechanism [38].

When habitat is lost from patches, our results produce the same counterintuitive extinction dynamics as existing models of competition–colonization coexistence—that the competitively dominant species goes extinct first [10]. However, our coexistence mechanism produces this result for a different reason. Intuition might have suggested that peripheral habitat loss would drive the most inferior competitor to extinction. Not only is this species inferior (in both dispersal and competitive abilities), but also it dominates the edge habitat that is being lost. However, for the dominant species to persist, it requires habitat that is distant from the patch boundary (since this represents a high source of mortality for it). As the patch shrinks, all of the remaining habitat becomes close to the edge, unsuitable for the dominant disperser. Thus, the loss of peripheral habitat will cause the extinction of the dominant, interior species (electronic supplementary material, figure S3).

The Earth's ecosystems contain an extraordinary diversity of species. A central challenge of coexistence theory is to identify the ecological differences that have allowed this diversity to be created and maintained. The geometry of a landscape can be easy to overlook when thinking about coexistence. It forms the backdrop against which the species interact, the domain within which physical and environmental factors create niches and facilitate coexistence. However, our mechanism shows that this landscape geometry could play an active role in maintaining species diversity, and that management choices about the convexity and complexity of a habitat boundary could make the difference between coexistence and exclusion, between persistence and extinction.

## Data Availability

MATLAB code used to generate the figures presented in this paper is available via the GitHub repository https://github.com/MikeBode/Spatial_coexistence. This repository includes the digitized outlines of the Kimberly archipelago islands. Supplementary material is available online [39].
